# Prognostic Relevance of HJURP Expression in Patients with Surgically Resected Colorectal Cancer

**DOI:** 10.3390/ijms21217928

**Published:** 2020-10-26

**Authors:** Dong Hyun Kang, Jongsoo Woo, Hyeongjoo Kim, Soo Youn Kim, Sanghee Ji, Gunn Jaygal, Tae Sung Ahn, Han Jo Kim, Hyoung Jong Kwak, Chang-Jin Kim, Moo-Jun Baek, Dongjun Jeong

**Affiliations:** 1 Department of Surgery, College of Medicine, Soonchunhyang University, 31 Soonchunhyang 6 gil, Dongnam-gu, Cheonan, Chungcheongnam-do 31151, Korea; c100048@schmc.ac.kr (D.H.K.); eyetoeye@schmc.ac.kr (T.S.A.); ssurge@sch.ac.kr (M.-J.B.); 2 Soonchunhyang Medical Science Research Institute, College of Medicine, Soonchunhyang University 31 Soonchunhyang 6 gil, Dongnam-gu, Cheonan, Chungcheongnam-do 330-722, Korea; jongsoo@sch.ac.kr (J.W.); khj@sch.ac.kr (H.K.); sooy.kim@sch.ac.kr (S.Y.K.); tkdgml675@naver.com (S.J.); gunjay@daum.net (G.J.); 3 Department of Oncology, College of Medicine, Soonchunhyang University, 31 Soonchunhyang 6 gil, Dongnam-gu, Cheonan, Chungcheongnam-do 31151, Korea; hzmd@schmc.ac.kr; 4 Research Institute of Clinical Medicine, Woori Madi Medical Center, 111 Baekjedae-ro, Wansan-gu, Jeonju, Jeollabuk-do 55082, Korea; k-h-jong@hanmail.net (H.J.K.); mountain48@hanmail.net (C.-J.K.); 5 Department of Pathology, College of Medicine, Soonchunhyang University, 31 Soonchunhyang 6 gil, Dongnam-gu, Cheonan, Chungcheongnam-do 31151, Korea

**Keywords:** Colorectal cancer (CRC), HJURP, prognostic biomarker

## Abstract

HJURP is a key factor for CENP-A deposition and maintenance in centromeres. The role of mis-regulation of histone chaperones in cancer initiation and progression has been studied. However, its role in colorectal cancer is still unclear. In this study, we aimed to evaluate the expression of HJURP in 162 colorectal cancer tissue. To investigate the function of HJURP in the colorectal cancer cell, we suppressed HJURP expression by siRNA and confirmed proliferation, migration, invasion, and anchorage independent of colony forming ability. The association between HJURP expression levels and clinicopathological factors was evaluated in 162 CRC tissues using immunohistochemistry. The overall survival rate in patients of HJURP high expression was higher than those in HJURP low expression in CRC. Suppressing HJURP expression decreased cellular proliferation, invasion, and migration in four CRC cell lines: HT29, HCT116, SW480, SW620 in vitro study. Our findings revealed that the knockdown of HJURP suppressed the proliferation, migration, invasion, and tumorigenicity in CRC cells. Due to its strong association with CRC, HJURP could be a potential prognostic biomarker and a novel target for drug discovery.

## 1. Introduction

Holliday Junction Recognition Protein (HJURP, also known as hFLEG1) is a centromeric protein with a pivotal role in the incorporation and maintenance of histone H3-like variant Centromere Protein-A (CENP-A) in centromeres. It is a specific chaperone for CENP-A and it integrates newly synthesized CENP-A molecules and nucleosomes at replicated centromeres [1]. Its centromeric localization is regulated by the cell cycle, and its brief appearance in the centromere coincides with new CENP-A deposition. Therefore, HJURP is a key factor for CENP-A deposition and maintenance in centromeres [2]. The role of mis-regulation of histone chaperones in cancer initiation and progression has been studied [3]. Furthermore, HJURP was reported to be overexpressed in lung cancer [4], and it has been proposed as an independent prognostic marker for luminal A breast carcinoma [5]. However, its role in colorectal cancer is still unclear.

Colorectal cancer (CRC) is one of the most common malignancies [6] and the leading cause of cancer-related death globally [7] with metastases as the main cause of mortality. Distant metastasis of CRC is present in approximately 25% of patients at diagnosis [8]. In distant metastatic CRC patients, median survival has improved from less than 10 months with best supportive care to 24 months even with chemotherapy [9,10,11,12,13,14]; however, mortality of CRC patients with distance metastasis is still poor. Therefore, early diagnosis and prediction of CRC metastasis is important to elevate a survival rate of CRC patients. Early detection of CRC, improved understanding of its clinicohistological prognostic factors, and its surgical treatment with or without chemotherapy or radiation therapy have contributed to the improved survival of affected patients. However, no marked variability in outcome exists that can predict the patients who have potential higher risk in recurrence, metastasis, resistance to chemotherapy, and decreased survival by using conventional histopathologic staging. Thus, identification of novel prognostic biomarkers in colorectal cancer is vital to develop effective targeted treatment for clinical benefits.

In this study, we conducted a pathologic evaluation of HJURP expression in CRC using immunohistochemistry (IHC), followed by a functional evaluation of HJURP knockdown by siRNA in four colorectal cancer cell lines to investigate prognostic biomarkers.

## 2. Results

### 2.1. Association Between HJURP Expression and Cancer-Specific Deaths in CRC Patients

**Figure 1:**
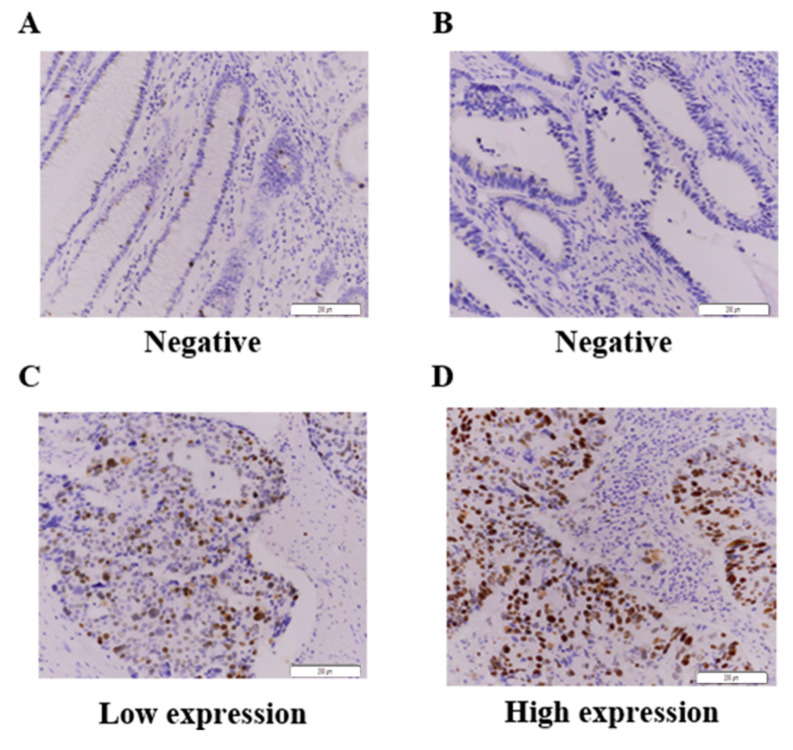
Immunohistochemistry for HJURP expression in colorectal cancer tissues. (**A**–**D**). One hundred and sixty-two CRC samples were stained with HJURP antibody and scored based on both staining intensity and staining frequency. Representative images of tissue slides and immunohistochemistry are shown ((**A**), Normal negative HJURP expression; (**B**), Tumor negative; (**C**), Tumor low expression; (**D**), Tumor high expression). Original magnification × 200.

IHC was used to assess HJURP expression in CRC specimens, and positive expression was found in the cytoplasm and/or cell membrane, as evident by brown staining (Figure 1). Overall, 162 cases of CRC specimens from patients were used. High HJURP expression was detected in 44 CRC tissues (27.2%), whereas 118 CRC tissues (82.8%) exhibited low expression of HJURP, as listed in Table 1. High HJURP expression was not significantly associated with pathologic factor. As shown in Figure 1, weak or no staining was observed in the normal epithelium, whereas moderate or strong immunostaining of HJURP was found in CRC tissues.

Stage, metastasis, lymphatic invasion, and HJURP expression had a significant impact on the 5-year survival of CRC patients, according to the univariate analysis. The multivariate Cox regression analysis adjusted for significant factors was used to examine the 5-year survival. Stage (HR = 1.771; 95% CI = 1.087–2.886; *p* = 0.022), metastasis (HR = 3.559; CI = 1.437–8.813; *p* = 0.006), lymphatic invasion (HR = 1.573; CI = 0.954–2.592; *p* = 0.076), and HJURP expression (HR = 1.880; CI = 1.167–3.027; *p* = 0.009) were the independent predictors of 5-year survival (Table 2). We also performed Cox-regression analysis by grouping patients positive and negative of HJURP expression. The results of the Cox-regression analysis was showed significant differences between the two groups (*p* < 0.05, Univariate; HR = 1.458; CI = 1.100–1.932; *p* = 0.009, Multivariate; HR = 1.724; CI = 1.032–2.880, *p* = 0.037). This suggests that the expression of HJURP decreases the survival rate of colorectal cancer patients.

**Figure 2:**
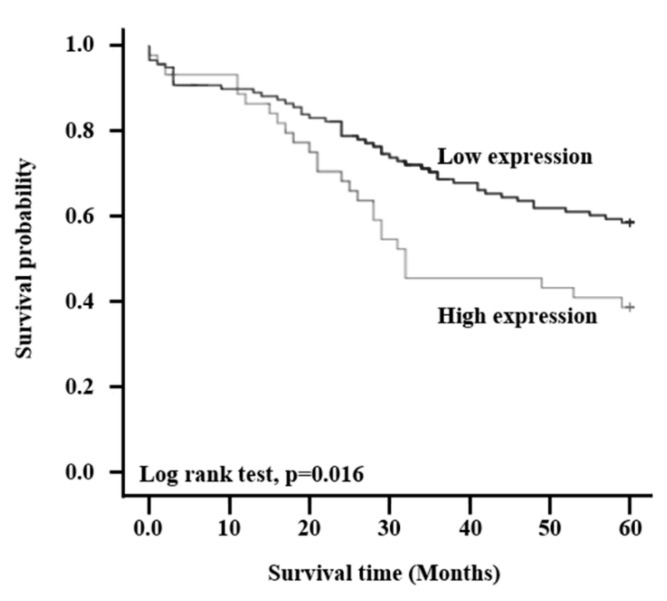
HJURP expression is related to overall survival in colorectal cancer patients. Patients were divided into two groups based on the expression of HJURP and survival rate was determined using Kaplan-Meier analysis (*p* = 0.016).

Kaplan-Meier estimates in the clinicopathological factors revealed that patients with high expression of HJURP had significantly reduced cancer-specific survival rates compared to those with low HJURP expression (log-rank test; *p* = 0.016), as shown in Figure 2.

### 2.2. Transduction of CRC Cells by siRNA

**Figure 3:**
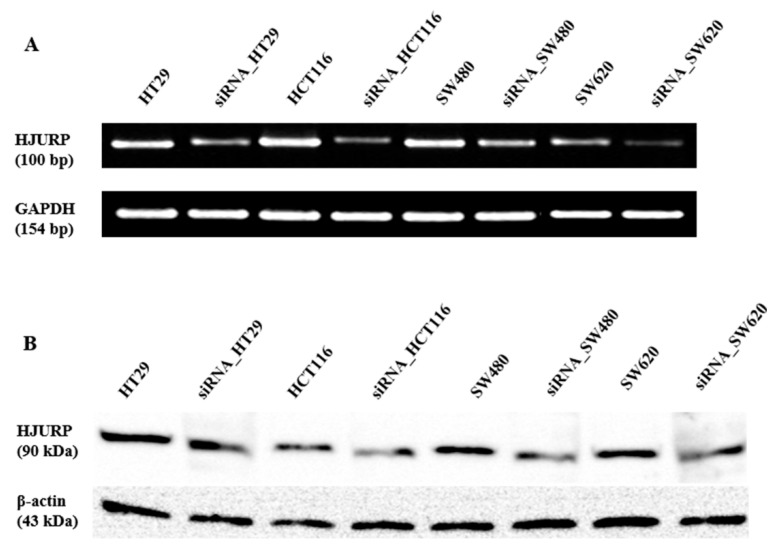
siRNA-mediated HJURP depletion in colorectal cancer cell lines. Colorectal cancer cell lines including HT29, HCT116, SW480, and SW620 were transduced with either control or HJURP siRNA. (**A**) RT-PCR was performed to determine HJURP mRNA expression cancer cell lines. (**B**) Immunoblot with anti-HJURP antibody was conducted to analyzed HJRUP protein level. GAPDH and β-actin was used as the loading control for RT-PCR and immunoblot.

To determine the role of HJURP expression in colon cancer, the experiment was performed. We examined the HJURP expression level in four CRC cell lines (HT29, HCT116, SW480, and SW620) and transfected these cells with siRNA to specifically target human HJURP mRNA. We confirmed the downregulation of HJURP expression using RT–PCR and Western blotting. Both HJURP mRNA and protein expression levels were barely detectable in HJURP–siRNA-transfected cells compared with that in control CRC cells (Figure 3).

### 2.3. Reduction of Colorectal Cancer Cell Proliferation by siRNA-Induced Downregulation of HJURP Expression

**Figure 4:**
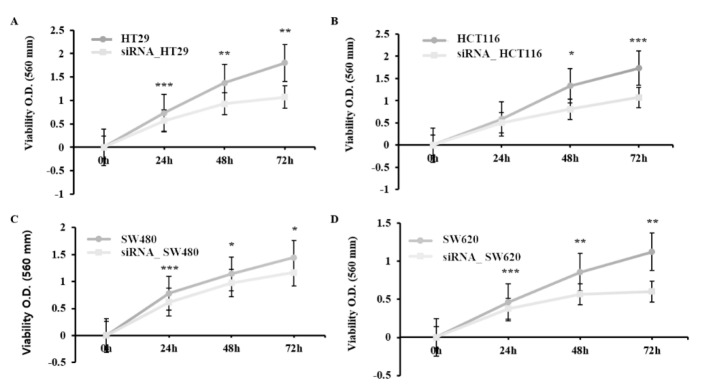
HJURP depletion impairs cell proliferation in colorectal cancer cell lines. Control or HJURP-siRNA colorectal cancer cell lines such as HT29, HCT116, SW480, and SW620 were analyzed every 24 h for 3 days to determine the proliferation rate by MTT assay. (**A**) HT29 cells. 24 h; *** *p* = 0.001, 48 h; ** *p* = 0.007, 72 h; ** *p* = 0.01, (**B**) HCT116. 48 h; * *p* = 0.021, 72h; *** *p* = 0.001, (**C**) SW480. 24 h; *** *p* = 0.001, 48 h; * *p* = 0.011, 72 h; * *p* = 0.021. (**D**) SW620. 24 h; *** *p* = 0.001, 48 h; ** *p* = 0.003, 72 h; ** *p* = 0.007.

To verify the effect of HJURP on cell proliferation, RNA interference against HJURP was performed. The proliferation of the transfected cells was shown by the MTT assay. Cells treated with HJURP target siRNA proliferated at a lower rate than the control cells (Figure 4; *p* < 0.005). The proliferation rates were compared: siRNA_HCT116 at 48 h (61%) and 72 h (62%); siRNA_HT29 cells at 24 h (77%), 48 h (68%), and 72 h (59%); siRNA_SW480 at 24 h (79%), 48 h (85%), and 72 h (81%); siRNA_SW620 at 24 h (83%), 48 h (66%), and 72 h (54%). These results suggest that the silencing of HJURP inhibits cell proliferation.

### 2.4. Suppression of Migration and Invasion of CRC Cells by Downregulation of HJURP

**Figure 5:**
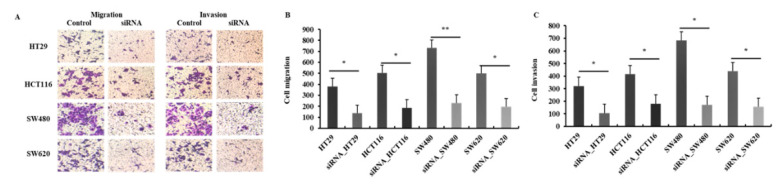
HJURP depletion impairs migration and invasion of colorectal cancer cell lines in vitro. Control or HJURP-siRNA colorectal cancer cell lines were seeded on a matrigel uncoated or coated Transwell, followed by incubation for 48 h for assessing migration and invasion, respectively. (**A**) Imaging was done using an inverted microscope and representative images are shown (× 40). (**B**) HJURP-siRNA cells displayed significantly reduced ability of migration (HT29; * *p* = 0.027, HCT116; * *p* = 0.023, SW480; ** *p* = 0.007, SW620; * *p* = 0.031). (**C**) siRNA mediated depletion of HJURP expression was suppressed the invasion ability of colorectal cancer cells (HT29; * *p* = 0.021, HCT116; * *p* = 0.028, SW480; * *p* = 0.011, SW620; * *p* = 0.026).

To further characterize the function of HJURP in cancer progression, a Transwell chamber assay was performed for cell migration and invasion. As shown in Figure 5, downregulation of HJURP expression in CRC cells significantly decreased cell migration and invasion compared with that in control cells. In the migration assay, groups treated with HJURP target siRNA showed significantly decreased migration (36.3 ± 3.27%) to the lower chamber compared with the control cells (*p* < 0.05). The four decreased rates were as follows: HT29, 36.3%; HCT116, 37.3%; SW480, 31.8%; and SW620, 39.6%. Downregulation of HJURP significantly inhibited cell invasion as compared with control cells with an approximate rate of 34.5 ± 7.96% (*p* < 0.05). The decreased rates were as follows: HT29, 33.3%; HCT116, 44.0%; SW480, 24.7%; and SW620, 36.2%. These results indicated that HJURP attenuates CRC cell migration and invasion.

### 2.5. CRC Cell Anchorage-Independent Growth Reduction by HJURP

**Figure 6:**
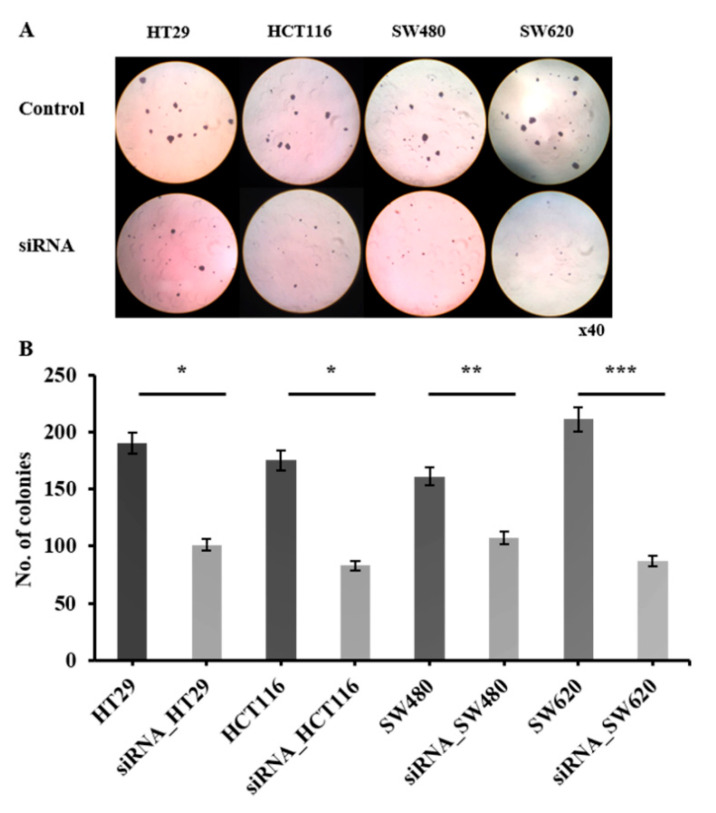
HJURP depletion impairs anchorage-independent colony forming ability of colorectal cancer cell lines in vitro. Control or HJURP-siRNA colorectal cancer cell lines were seeded on soft agarose and incubation was done for 14 days to evaluate colony forming ability. (**A**) Representative images of colonies were shown (× 40). (**B**) Number of colonies from each colorectal cancer cell lines were quantified (HT29; * *p* = 0.038, HCT116; * *p* = 0.05, SW480; ** *p* = 0.008, SW620; *** *p* = 0.001).

To determine whether HJURP is required in anchorage-independent growth, one of the hallmarks of oncogenic transformation (anchorage and growth regulation in the normal and virus-transformed cell), soft agar colony formation assay was performed. HJURP target siRNA transfected cells showed a significant reduction in colony formation efficiency of 52.9 ± 10.15% (*p* < 0.05; 95.1 ± 8.41 colonies). The colony numbers of the four cell lines are as follows: HT29, 190 ± 7.51 and 102 ± 8.02; HCT116, 175 ± 5.51 and 87 ± 3.61; SW480, 161 ± 9.00 and 107 ± 8.50; and SW620, 211 ± 6.51 and 89 ± 9.61, for the control group and siRNA-treated group, respectively (Figure 6). These findings indicate that HJURP is an important participant in CRC cell anchorage-independent growth.

## 3. Discussion

CRC was the third most commonly diagnosed cancer (affecting 1.36 million individuals) worldwide in 2018, as reported by GLOBOCAN [15]. The TNM staging system is mainly used to determine CRC prognosis but is not sufficiently reliable because of the cellular heterogeneity of the tumors. Therefore, novel prognostic methods and predictive biomarkers should be developed to improve prognosis prediction and determine the treatment plan.

HJURP is a key factor for the deposition of histone H3 variant CenH3 in centromeric chromatin to maintain proper chromosome segregation. It plays an important role in various human malignant tumors, such as lung cancer [4], breast cancer [5], and hepatocellular cancer [16,17]. However, its function in colorectal cancer has yet to be discussed.

Our findings are from in vitro studies on the function and significance of HJURP expression in the progression of malignancy. HJURP functions are related to the deposition of CENP-A in the centromere, and its activation is required for chromosomal stability. It is also involved in immortalizing cancer cells [4,18,19], and its abnormal upregulation has been reported in several human cancers [4,5,16,17,20].

HJURP interacts with CENP-A to localize CENP-A and load new CENP-A nucleosomes on the centromere as its specific chaperone [1,2]. Though CENP-A is a histone H3 variant, unlike canonical histone H3, its chromatin binding is not associated with DNA replication in vertebrate cells [21]. Newly synthesized CENP-A is incorporated into centromeres, specifically in the early G1 phase in human cells [21].

CENP-A is the key player in centromere formation and kinetochore assembly. It performs a complex job of attaching chromosomes to the mitotic spindle and ensures that those attachments are correct. Thus, the dysregulation of CENP-A delays the signaling in mitotic progression and the regulation of chromosome movements towards the spindle poles in anaphase. In human breast cancer, overexpression of HJURP may be similar to overexpression of mitotic kinases, such as Aurora kinases, which induce genomic instability, one of the hallmarks of tumor development [21].

The molecular mechanism of HJURP in the oncogenesis of CRC has not yet been elucidated. However, Chen et al. [16] reported that HJURP modulates the cell cycle by regulating p21, a potent cyclin-dependent kinase inhibitor (CKI), ERK1/2, and GSK3β. Because the nuclear localization of p21 is mediated by the MAPK/ERK1/2 pathway in CRC, the oncogenic function of HJURP in CRC may also be suggested by the same pathway. The precise mechanisms of HJURP oncogenic function should be studied further.

Here, we showed that high HJURP expression was not associated with clinicopathological factors. However, we found that the overall survival is poor in patients with lymph nodal metastases, nerve invasion, metastasis, and high HJURP expression using multivariate Cox regression analysis. Additionally, Kaplan-Meier analysis revealed that high HJURP expression was correlated with poor overall survival rates in CRC patients. Analyses of The Cancer Genome Atlas (TCGA) databases revealed that 5-year survival rate of patients HJURP high expression is 55%, while that of HJURP low expression is 64%. The difference of 5-year survival between two groups is only 9% without statistical significance.

We also demonstrated that overexpression of HJURP was inversely associated with CRC prognosis, and thus can be used as an independent prognostic factor in CRC patients.

We focused on its expression in CRC tissues and the effects of its downregulation by siRNA on four CRC cell lines in terms of cell proliferation, migration, invasion, and colony formation in vitro. siRNA also suppressed the proliferation, invasion, and migration abilities of the four cell lines. Tumorigenicity in vitro, as evaluated by soft agar colony formation, was reduced notably after transfection with siRNA. These results suggest that HJURP acts as an oncogene in CRC.

## 4. Materials and Methods

### 4.1. Cell Lines and Culture

Human colorectal cell lines, including HCT116, HT29, SW480, and SW620, were purchased from the Korean Cell Line Bank (KCLB, Seoul, Korea). Each was propagated in RPMI1640 (ThermoFisher, Waltham, MA, USA) and supplemented with 10% fetal bovine serum (FBS, Thermofisher) and 1% penicillin/streptomycin (Gibco by life technologies, Thermo Fisher Scientific, Waltham, MA, USA)) in a fully humidified incubator containing 5% CO_2_ at 37 °C.

### 4.2. Silencing of HJURP by siRNA

The Specific siRNA for HJURP was purchased from OriGene (Rockville, MD, USA, #SR310705). Approximately 1 × 10^5^ cells per well were seeded in 6-well plates and incubated for 24 h, then culture with serum-free medium 12 h before the transfection. Then, the cells were transfected HJURP-siRNA using HiPerFect transfection reagent (Qiagen, IN, USA, #301705) in serum-free medium, according to the instructions provided by the manufacturer. Thirty-six hours after transfection, cells were collected for RNA and protein isolation and evaluation for the HJURP knockdown. The experiments were performed in triplicate.

### 4.3. Reverse Transcriptase PCR

Total RNA was extracted using RioEx reagent (Geneall, Seoul, Korea) from the cultured cells. cDNA was reverse-transcribed from total RNA using a ReverTra Ace qPCR kit (Toyobo, Osaka, Japan) according to the manufacturer’s instruction, and GAPDH was used as an internal control. The PCR amplification program employed was: pre-denaturation at 94 °C for 5 min, denaturation at 94 °C for 1 min, annealing of HJURP and GAPDH performed at 58 °C for 30 s, and a final extension performed at 72 °C for 1 min. A total of 35 cycles was performed. The oligonucleotide primers used were as follows: HJURP forward, 5′-CAGATGGGGTGGACAACAC-3′; HJURP reverse, 5′-TCTTCCATCCTGTAAGACGTG-3′; GAPDH forward: 5′-CTTAGCACCCCTGGCCAAG-3′; GAPDH reverse: 5′- GATGTTCTGGAGAGCCCCG-3′. The expected PCR product sizes were HJURP 100 bp and GAPDH 154 bp. The PCR products were electrophoresed on a 2% agarose gel and visualized using ethidium bromide staining for 10 min. We performed all analyses in triplicate.

### 4.4. Western Blotting Assay

The cells were lysed in Pro-PREP^TM^ protein extraction solution (INtRON, Seongnam, Korea). Proteins were quantified (30 μg/lane) by bicinchoninic acid (BSA, Sigma, Yongin, Korea) assay using xMark Microplate Absorbance Spectrophotometer (Bio-Rad Laboratories, Inc., Hercules, CA, USA). The proteins were resolved by 10% sodium dodecyl sulfate–polyacrylamide gel (SDS–PAGE) electrophoresis and transferred to Immobilon polyvinylidene difluoride membranes (Millipore, MA, USA). The transferred membranes was blocked in 5% skim milk for 1 h and were incubated in anti-human HJURP polyclonal antibody (Sigma-Aldrich, St. Louis, MO, USA) diluted at 1:300 overnight at 4 °C, followed by another incubation with secondary antibody diluted at 1:2000 for 2 h at room temperature. Signal was detected by ECL western detection reagents (Advansta, San Jose, CA, USA) through Molecular Imager ChemiDoc XRS+ System (Bio-Rad Laboratories, CA, USA).

### 4.5. Cell Proliferation Assay

The 3-(4,5-dimethlthiazol-2-yl)-2,5-diphenyltetrazolium bromide (MTT) assay was performed in 96-well (3 × 10^4^ cells/well) culture plates. The cells were incubated for 24, 48, and 72 h at 37 °C and 5% CO_2_. Afterward, 10 μL MTT (5 mg/mL) was added to the appropriate wells, followed by a 2 h incubation. The supernatant was then discarded, and 100 μL of dimethyl sulfoxide (DMSO) was added to each well to dissolve the precipitate. The viability was quantified by measuring its absorbance at 570 nm.

### 4.6. Cell Invasion and Migration Assay

Cell invasion and migration assay was performed using Transwell Permeable Supports 6.5 mm Insert (Cosar, Corning, NY, USA) and growth factor reduced Matrigel (BD Biosciences, San Jose, CA, USA). The supports were incubated for 1 h at 37 °C and covered with Matrigel. Cells at a density of 5 × 10^5^ cells/well were then added, and 750 μL culture medium with 10% FBS was added to the bottom chamber. After incubation for 48 h, the media was removed from Transwell, and fixed cell with 3.7% paraformaldehyde after washing with cold PBS. The cells were stained with 0.5% crystal violet. Matrigel in the upper membrane was removed. The number of cells was counted using a microscope. Migration assay was performed in a similar manner but without using Matrigel.

### 4.7. Soft Agar Colony Formation Assay

Six-well plates were coated with 0.5% agarose in RPMI1640 media supplemented with 20% fetal bovine serum (FBS). The HJURP knockdown cells or control cells (each cell number, 2.5 × 10^3^ cells/mL) were mixed in RPMI1640 media containing 0.35% agar and overlaid on 0.5% agar in 6-well plate. The plates were incubated for 14 days at 37 °C with 5% CO_2_ until colonies were formed. Afterward, each well was stained with 0.01% crystal violet, and the number of colonies was counted and photographed.

### 4.8. Immunohistochemistry (IHC)

A total of 162 samples were collected from patients who underwent surgery and were histopathologically confirmed to have CRC at Soonchunhyang University, Cheonan Hospital between January 2002 and December 2009. Patient data are presented in Table 1. This study was approved by the Ethics Committee of Soonchunhyang University, Cheonan Hospital.

Paraffin-embedded blocks of CRC tissues were cut into 5 μM thick sections. The slides were retrieved and incubated overnight at 4 °C with human HJURP antibody (Sigma-Aldrich, St. Louis, MO, USA). They were then washed in Tris-buffered saline with Tween^®^ 20 (TBST) and incubated at room temperature for 30 min with a secondary antibody using EnVision+ System- HRP Labelled Polymer Anti-Rabbit (Dako, CA, USA). The slides were treated with 70 μL 3′-3-diaminobenzidine (DAB, Dako, CA, USA) and evaluated using a microscope. Immunohistochemical parameters were graded by three independent investigators in a blinded manner, according to a semi-quantitative optical analysis, and a consensus score was decided for each specimen. Quantification of HJURP was performed by assessing the percentage and intensity of the stained tumor cells. The staining percentage was scored as follows: 0, 0–5%; 1, 5–25%; 2, 25–50%; 3, 50–75%; and 4, 75–100%, whereas staining intensity was scored in the following manner: 0, negative; 1, weak; 2, moderate; 3, strong. The final scores were then grouped into low or high expression groups (low: scores < 2; high: score ≥ 2).

### 4.9. Statistical Analysis

Statistical analysis was performed through the SPSS v19 program using Student’s *t*-test. 95% confidence interval (CI) and hazard ratio (HR) of clinicopathological variables were evaluated using univariate and multivariate Cox regression models. Kaplan-Meier method was used to analyze the associations between HJURP expression and the outcome of patients. The log-rank test was used to assess the overall survival rate. For all tests, a *p* value < 0.05 was statistically significant

## 5. Conclusions

Our findings revealed that the knockdown of HJURP suppressed the proliferation, migration, invasion, and tumorigenicity in CRC cells. Due to its strong association with CRC, HJURP could be a potential prognostic biomarker and a novel target for drug discovery. Efficient inhibition of HJURP is a possible subject for cancer research.

## Figures and Tables

**Table 1:** Clinicopathological features in patients with colorectal cancer.

Clinicopathological Factors	HJURP Expression		Total (*N* = 162)	*p* Value
	Low (*N* = 118)	High (*N* = 44)		
**Age, years, mean (SD)**				0.240
**Gender, N (%)**				0.042
**F**	41 (34.7)	23 (52.4)	64 (39.5)	
**M**	77 (65.3)	21 (47.7)	98 (60.5)	
**pT stage, N (%)**				0.541
**1 and 2**	20 916.9)	7 (15.9)	27 (16.7)	
**3 and 4**	98 (83.1)	37 (84.1)	135 (83.3)	
**pN stage, N (%)**				0.797
**negative**	59 (50.0)	23 (52.3)	82 (50.6)	
**positive**	59 (50.0)	21 (47.7)	80 (49.4)	
**Metastasis, N (%)**				0.729
**negative**	114 (96.6)	42 (95.5)	156 (96.3)	
**positive**	4 (3.4)	2 (4.5)	6 (3.7)	
**Vascular invasion, N (%)**				0.891
**negative**	95 (80.5)	35 (79.5)	130 (80.2)	
**positive**	23 (19.5)	9 (20.5)	32 (19.8)	
**Lymphatic invasion, N (%)**				0.677
**negative**	87 (73.7)	31 (70.5)	118 (72.8)	
**positive**	31 (26.3)	13 (29.5)	44 (27.2)	
* Stage, N (%)				0.797
**I and II**	59 (50.0)	23 (52.3)	82 (50.6)	
**III and IV**	59 (50.0)	21 (47.7)	80 (49.4)	

* AJCC Cancer Staging Manual, Eighth Edition (2017).

**Table 2:** Cox regression analysis of the clinicopathological parameters in colorectal cancer patients.

Clinicopathologic Factors	Variable	Univariate Analysis		Multivariate Analysis	
		Hazard Ratio (95%/CI)	*p* Value	Hazard Ratio (95%/CI)	*p* Value
**Age**	<60 yr vs. ≥60 yr	0.990 (0.607–11.615)	0.967		
**Gender**	Female vs. Male	0.864 (9.549–1.360)	0.528		
**Stage**	I, II vs. III, IV	1.974 (1.245–3.130)	0.004	1.771 (1.087–2.886)	0.022
**Metastasis**	Negative vs. Positive	6.000 (2.522–14.272)	0.000	3.559 (1.437–8.813)	0.006
**Vascular Invasion**	Negative vs. Positive	1.516 (0.893–2.575)	0.124		
**Lymphatic Invasion**	Negative vs. Positive	2.026 (1.2264–3.247)	0.003	1.573 (0.954–2.592)	0.076
**HJURP Expression**	Low vs. High	1.766 (1.103–2.829)	0.018	1.880 (1.167–3.027)	0.009

## Abbreviations

CRC	Colorectal cancer
HJURP	Holliday Junction Recognition Protein
CENP-A	Centromere Protein-A
IHC	Immunohistochemistry
siRNA	Small interfering RNA
HR	Hazard ratio
CI	Confidence Interval
TNM	Tumor-Node-Metastasis
CKI	Cyclin-dependent kinase inhibitor
ERK	Extracellular Signal Regulated Kinase
GSK3β	Glycogen Synthase Kinas 3 Beta
MAPK	Mitogen-Activated Protein Kinase
